# Supplementary material for: Is it really a piece of cake to label Geographical Indications with the Nutri-Score? Consumers’ behaviour and policy implications
Source: PLoS One. 2022 Nov 17;17(11):e0277048. doi: 10.1371/journal.pone.0277048 (PMC9671382; doi:10.1371/journal.pone.0277048)
Supplement: Attachment — Submitted filename: Response to the Revierers comments.docx [file pone.0277048.s001.docx]

In the following pages you can find our point-by-point responses to your comments. Each Reviewer Comment (**RC**) will be followed by our Answer (**A**), reported in blue. In the main document, modifies are highlighted in yellow.

**REVIEWER 1:**

Dear Authors, I appreciate your research. In my opinion, it's well written. The data support the conclusions and the statistical analysis has been performed appropriately but I have some revisions to suggest.

**RC_1:** The sample is not representative, and I think you should better specify the selection criteria and modalities. **A_1:** Thank you for your comments. The sample of the present study is stratified and assures the representativeness of the data according to the following criteria: geographical distribution of the respondents, gender and age. Following you suggestion, we better specify this aspect in the paper (page 5).

**RC_2:** In addition, in some parts I think the literature is a bit outdated (e.g. p.9 line 37 the reference is to 2008).

**A_2:** According to your comment, we have updated the literature. As it is referred to the traditional labeling (i.e., the Back of Pack ones), literature is a bit outdated in this point. However, following your suggestion, we have better specify this point in the text (page 2).

**RC_3:** I suggest moving then table 7 to the results part and not the discussions.
I would like you to include some more managerial implications for Italian companies in the light of what is happening abroad.

**A_3:** Following your comment, we have moved table 7 in the results part.

With regard the second point of your comment, to the best of our knowledge, there are no studies that aims to understand the impact of Front-Of-Pack nutritional label – and specifically of Nutri-Score – on the consumption of GI products. It follows that a comparison with what is happening abroad for different type of food could be tricky. However, we discussed some implication of the use of the NS on generic cheese, as you can see in the conclusions.

**REVIEWER 2:**

Looking at the interaction between the influences of different types of labels is an interesting topic that should be of interest to many readers. I do though have some comments and questions on the manuscript that are detailed below. I hope they are helpful.

**RC_1:** I’d probably work on a different title. The ‘cake and eating it’ line in conclusion feels forced.

**A_1:** Thank you for your comment. According to your suggestion, the phrase ‘cake and eating it’ has been removed from the conclusions and the concept has been explained in a more explicit way.

**RC_2:** There seem to be some simple writing errors, such as in the first sentence of the abstract, where I assume “UE” is supposed to be “EU.” If not, what is UE?

**A_2:** Thank you for your comment. We have corrected the typo in the text.

**RC_3:** I’m concerned with the idea of equating promoting GI’s with promoting an unhealthy policy. GI’s as you know are not based on healthiness criteria and certainly that isn’t the goal behind the program. So, I wouldn’t push too hard this narrative that they are contrasting policies when they are really policies with very different goals and reasons for being

**A_3:** We agree with you that the GI policy does not promote a healthier lifestyle. However, in our opinion, the compulsory application of Nutri-Score on GI products, as foreseen by F2F strategy, may generate contrasting effects. Let's try to explain ourselves better: at consumer behavior level, the policy behind the GI supports the consumption of PDO and PGI products, which are certified and protected for their superior quality. On the contrary, within the F2F strategy, NS could penalize most of the GI products of animal origin, as they are considered unhealthy. Thus, there would be two policies that incentivize consumers to implement two opposite behaviors, even if such policies have different aims.

**RC_4:** P. 3. So are you saying the EU’s goal is to get consumers to only buy healthy products? Are you proposing that GI’s are abandoned since they mostly ‘help’ unhealthy foods?

**A_4:** Thank you for your comment. Based on your questions, we have re-written the abstract for better specify our opinion. In our work, we are stressing that GIs could be penalized by EU adoption of the NS, as most of them could be considered as products to be avoid or to be reduced in consumption. The EU policy should not only focused in promoting healthy products, but it is supporting the quality in a “holistic” way. With the aim of reducing the information asymmetry, the two policies (F2F and GI) are providing consumers with additional information, helping them to choose what to buy in a conscious way. However, the information provided are, in some way, contrasting. On one hand, the GI logo on food products means “high quality goods”, thus incenting the sales of GIs. On the other hand, the F2F are considering the most of GIs as products to be avoid or to be reduced in consumption for healthy reason, thus discouraging the purchase of these products. In light of this, we think that there is a clear need to better investigate this issue at EU level, considering also the negative impact of the NS on GIs. As proposed by many authors, to avoid potential misinterpretation, it would be useful to synchronize the introduction of such a scheme with an educational campaign to inform consumers about how to correctly interpret the information on food labels.

**RC_5:** As I’m not familiar with the Nutri-Score system I’d like a bit more detail how this single measure is created (I’m more familiar with the MTL). Maybe some more on its strengths/weaknesses relative to the MTL. Maybe some examples of different ratings on GI products?

**A_5**: According to you suggestion, we have better detailed the differences between the NS and the MTL, especially in the case of GI products (introduction part, page 4). Furthermore, we have linked the French Public Health website, which explains in depth how to calculate the NS for the different product categories.

**RC_6:** P. 4. L. 111. Be careful with phrasing, you aren’t asking if they are willing to pay for the label, but for the labeled product.

**A_6:** Thank you for your advice. The paper has been improved in this direction.

**RC_7:** I’d like more details on the “external agency” – the group doing this should not be secret.

**A_7:** According to your suggestion, we have reported the name of the agency in the paper (see page 5). The external agency is the Nortstat panel agency (<https://norstat.it>), headquartered in Norway and with offices in 11 other European countries, including Italy (based in Milan).

**RC_8:** Not clear that Figure 1 adds much beyond the text.

**A_8:** Thank you for your comment. Although it does not add additional info to the text, in our opinion Figure 1 could be useful to better visualize the experimental design.

**RC_9:** P. 5. Why are you using such wide Likert scales?

**A_9:** The scale used is 7-point Likert scale. The 9 reported in the text was a typo. Sorry for that.

**RC_10:** On my copy, there are formatting issues with Table 1.

**A_10:** Thank you for reporting that. We have formatted the table.

**RC_11:** Not sure Table A.1 is needed either.

**A_11:** We accepted your suggestion. Table A.1 was removed.

**RC_12:** P. 14, L. 338. I’d like more discussion and ideas behind this unexpected result. Certainly looks like the Nutri-Score doesn’t work well without education behind it.

**A_12:** Thank you for your comment. In this section we have only reported the results, which are better discusses in the following section. You can find it at lines 433-449.

**RC_13:** P. 16. Again, why are some of these results so different?

**A_13:** Thank you for your comment. Also in this case, such differences are discussed in section 4, and, specifically, at lines 468-486.

**RC_14 / RC_15:** The conclusions seem broad and overly cast GI’s as being unhealthy (GI is not equal to unhealthy). Towards the end, the paper appears to be more about the Nutri-Score and its abilities and less about its contrast with PDO’s. I wrote a note on my copy asking what the paper was really about.

**A_14/ A_15:** Thank you for your comment. We understand your point of view. As previously discussed (see point 3-4), we improve the conclusions trying to clarify that the F2F strategy applied on GI products could mislead consumers. Following your suggestion, we have added some parts in the conclusion, to better specify our point of view.

**RC_16:** I agree with the limitation of only looking at letter D scored products, especially considering my previous comment about how much the paper seems to be examining the value of the Nutri-Score system. It would be very helpful to know how people respond with some of the other grades – including finding GI’s that aren’t D.

**A_16:** We agree. As reported by Hafner and Pravst (2021), the application of the NS on cheeses is a concern in many countries. Indeed, only a few of these products (generic cheeses and not PDO products) can have a positive NS (grade A or B). It follows that consumers may consider cheeses as unhealthy foods. The lack of variability in the NS grade in this category could mislead consumers, as they are no longer able to distinguish the healthier option, considering that the aim of the NS is to guide consumers towards the healthiest choice comparing products of the same category. When it comes to the GI cheeses, as stressed in the paper, there are no products labelled with positive NS grade. Further studies should focus on understanding the effect of the NS on GI products, also using positive grades, applying the labelling on a wide products’ portfolio.

**RC_17:** You also have more limitations than you’ve noted. Think about these more. Also, what studies should be done next?

**A_17:** Following your suggestion we have added a limitation in the study, considering that it could not have external validity. We also added another possible study to be implement.
